# Downregulation of DUSP9 Promotes Tumor Progression and Contributes to Poor Prognosis in Human Colorectal Cancer

**DOI:** 10.3389/fonc.2020.547011

**Published:** 2020-09-23

**Authors:** Zhaoyan Qiu, Ning Liang, Qian Huang, Tao Sun, Hongyuan Xue, Tianyu Xie, Xinxin Wang, Qian Wang

**Affiliations:** ^1^Department of General Surgery, The First Medical Centre, Chinese PLA General Hospital, Beijing, China; ^2^Department of General Surgery, The 75th Group Army Hospital, Dali, China; ^3^Department of Obstetrics and Gynecology, The 75th Group Army Hospital, Dali, China; ^4^Departmentof Neurosurgery, First Affiliated Hospital, Zhengzhou University, Zhengzhou, China; ^5^Department of General Surgery, Huashan Hospital, Fudan University, Shanghai, China; ^6^Department of Anorectal Surgery, First Affiliated Hospital, Zhengzhou University, Zhengzhou, China

**Keywords:** DUSP9, colorectal cancer, CpG island, bisulfite sequencing, prognosis

## Abstract

**Background:**

Dual-specificity phosphatase 9 (DUSP9) belongs to the dual-specificity protein phosphatase subfamily. Recently, increasing attention has been paid on the role of DUSP9 in a variety of cancers. However, its functional role in tumor development is still unclear, especially in colorectal cancer (CRC).

**Methods:**

The functional role of DUSP9 in inhibiting the progression of CRC was verified using colony formation assay, wound healing assay, nude mice xenograft model, etc. RNA-seq was performed to assess the gene expression profiling in SW480 cells with DUSP9 stable knockdown and shControl cells. Bisulfite sequencing (BSE) was performed to reveal the methylation status of CpG island in the promoter of DUSP9.

**Results:**

DUSP9 was significantly downregulated in tumor tissues compared with peritumor tissues. Mechanistically, the high methylation status of CpG island in the promoter of DUSP9 may lead to the downregulation of DUSP9 in CRC. Clinically, low DUSP9 expression in CRC was closely associated with depth of invasion, metastasis (TNM) stage, and poor survival, indicating that DUSP9 may be involved in the progression of CRC. Functional study revealed that DUSP9 inhibited proliferation, migration, invasion, and epithelial–mesenchymal transition of CRC cells both *in vitro* and *in vivo*. Transcriptome profiling studies revealed that Erk signaling was involved in the tumor progression mediated by DUSP9 silencing, which is confirmed by cell experiments and clinical tissue sample staining analysis.

**Conclusion:**

Our findings demonstrate that DUSP9 plays a critical role in the progression of CRC, and therapeutic intervention to increase the expression or activity of DUSP9 may be a potential target for CRC treatment in the future.

## Introduction

Colorectal cancer (CRC) is a malignancy with high incidence in digestive system. According to statistics, there are about 1.2 million new cases and 600,000 deaths in the world every year (1). With the changing of population and dietary habit throughout the world, the number of new cases is on the rise every year (2). The recognized risk factors for CRC include dietary factors (such as high animal fat, high animal protein, high energy, and low cellulose diet with more westernized lifestyle), genetic factors (especially familial adenomatous polyposis and hereditary non-polyposis), and disease factors (such as colorectal adenoma, ulcerative colitis, and polyposis) (3). At present, surgical resection, radiotherapy, chemotherapy, and biotherapy are the main treatments, but the effect is very limited (4). Despite significant improvements in these treatments, CRC remains the second leading cause of cancer-related death (5). Moreover, the specificity and sensitivity of serum markers (such as CEA and CA199) in the diagnosis of colon cancer are difficult to achieve satisfactory results (4). Therefore, it is very important to screen and identify new key molecules that are involved in the initiation and progression of CRC.

Dual-specificity phosphatase 9 (DUSP9), also known as MKP-4 (mitogen-activated protein kinase phosphatase 4), belongs to the dual-specificity protein phosphatase subfamily, which has been reported to dephosphorylate tyrosine and threonine/serine residues of their substrates (6). Recently, it was observed that DUSP9 plays a critical role in the normal function of placenta, whereas is not required for mammalian embryonic development (7). In addition, Ye et al. (8) demonstrated that DUSP9 inhibits the progression of non-alcoholic fatty liver disease (NAFLD) through ASK1 suppression, suggesting that Dusp9 may be an ideal therapeutic target for the treatment of NAFLD. Recently, increasing attention has been paid on the role of DUSP9 in a variety of cancers. Accumulating evidence suggests that DUSP9 is downregulated and acts as a tumor suppressor in many kinds of cancers, such as gastric cancer, hepatocellular carcinomas, renal cancer, squamous cell carcinoma (SCC), etc. (9–12). Moreover, in a mouse model, DUSP9 has also been found to play an anti-tumor role in SCC and non-small cell lung cancer (NSCLC) (13). However, as far as we know, the expression pattern of DUSP9 in CRC has not been reported. Additionally, the function of DUSP9 in CRC and its regulatory mechanism are still unclear.

In the present study, we systematically investigated the expression and functional roles of DUSP9 in CRC as well as its clinical implication. This study provides a new dimension to understand the pathological roles of DUSP9 in CRC development and provides experimental evidence for DUSP9 as a potential therapeutic target in CRC.

## Materials and Methods

### Cell Culture and Tissue Collection

Human CRC cell lines SW480 and LoVo were purchased from ATCC (Manassas, VA, United States), cultured in RPMI-1640 medium (Gibco) containing 10% serum. The medium contained 100 U/ml penicillin and 100 μg/ml streptomycin. A total of 245 paired CRC tissues were collected at the Chinese PLA general hospital in the present study, which was approved by the Ethics Committee of the Chinese PLA general hospital. Moreover, all participants involved in this study have signed informed consent. For the clinical data, please see Table 1.

**TABLE 1:** Relationship between tumor DUSP9 expression and clinic features.

Variables	Number of cases	DUSP9 expression		*P*-value
		Low	High	
All	245	154	91	
Age				0.791
<60	117	75	42	
≥60	128	79	49	
Gender				0.792
Female	113	70	43	
Male	132	84	48	
Tumor site				0.181
Colon	142	84	58	
Rectum	103	70	33	
Tumor size				0.001
<5.0 cm	115	60	55	
≥5 cm	130	94	36	
Differentiation grade				0.219
Well	154	92	62	
Poor	91	62	29	
Depth of invasion				**0.003**
T1 + T2	112	59	53	
T3 + T4	133	95	38	
Lymph node metastasis				0.227
Absent	107	97	50	
Present	138	57	41	
Distant metastasis				0.409
Absent	157	102	55	
Present	88	52	36	
TNM stage				**0.005**
I + II	108	57	51	
III + IV	137	97	40	

*Bold indicates statistical significance.*

### Cell Viability Assay

MTS assay (G3581; Promega Corporation, Madison, WI) was used for cell viability detection. The procedure was performed as previously described (14). Briefly, RPMI (100 μl) was supplemented with MTS solution 20 μl/well, incubated for 2 h, and then the absorbance was measured at 490 nm using a spectrophotometer.

### Colony Formation Assay

Colony formation assay was performed as previously described (15). The stable transfected CRC cells (SW480 and LoVo) were seeded in six-well plates with the density of 1 × 10^3^/well. After 2 weeks, CRC cells were fixed in 70% ethanol and then stained with 5% crystal violet. Count the colony numbers under the microscope.

### EdU Incorporation Assay

EdU incorporation assay was performed according to the method previously described (16). The EDU reagent was diluted to 5 μm in serum-free medium and added to the cells for 2 h. After PBS cleaning, 4% paraformaldehyde was added for 30 min and then 0.5% Triton X-100 was added for 20 min. Dye these cells with Apollo^®^ reaction cocktail according to the instructions. The prepared Hoechst 33342 solution was added to stain the nucleus for 30 min. Finally, positive cells were counted.

### Wound Healing Assay

Wound healing assay was performed to assess cell migration ability (17). In short, three scratch wounds in each well were made using plastic pipette tips, and the wound closure was observed at 0 and 48 h.

### Nude Mice Xenograft Model

Six- to eight-week-old BALB/c nude mice were randomly divided into experimental group and control group. The establishment of mice xenograft model refers to previous methods (18). The calculation formula of tumor volume is as follows: *V* = 1/2 × *L* × *W*^2^ (*L*, the longest dimension; *W*, shortest dimension). This study was approved by the Ethics Committee of Chinese PLA general hospital for animal research.

### Western Blot and Immunohistochemical Staining

Western blot and immunohistochemistry staining were performed as previously described (19). For western blot assay, the primary antibodies against DUSP9 (1:2000, Abcam, cat. no. ab167080), E-cadherin (1:2000, Abcam, cat. no. ab194982), ZO-1 (1:1500, Abcam, cat. no. ab221547), Vimentin (1:2000, Abcam, cat. no. 92547), N-cadherin (1:1000, Abcam, cat. no. ab18203), and β-actin (1:3000, Abcam, cat. no. 179467) were used in accordance with the manufacturer’s instructions. Signals were detected using an ECL kit (Pierce, Rockford, IL) as previously reported (19). For immunohistochemistry staining, primary antibodies against DUSP9 (1:200, Abcam, cat. no. ab167080) and PCNA (1:1000, Abcam, cat. no. ab92552) were used according to the manufacturer’s instructions. The immunohistochemical (IHC) scoring methods were as follows: (1) the staining intensity of positive cells (recorded as A): 0 for non-staining, 1 for light staining, 2 for moderate staining, and 3 for strong staining; (2) the percentage of positive cells (recorded as B); 0 for 0–10%, 1 for 11–25%, 2 for 26–50%, 3 for 51–75%, and 4 for more than 75%; (3) the product of A and B is the final IHC score. Semi-quantitative analysis method: 0–1 for −, 2–4 for +, 5–8 for ++, and 9–12 for +++.

### RNA Extraction and Quantitative Real-Time PCR

Total RNA was extracted from tissues and cell lines using an RNA extraction kit (Takara, Tokyo, Japan). The RNA sample was reverse transcribed into cDNA using a reverse transcription kit (Takara, Tokyo, Japan). DUSP9-, GAPDH-, E- cadherin-, ZO- 1-, Vimentin-, and N-cadherin-specific primers were designed and synthesized by Takara. The primers are listed in Supplementary Table 4. The quantitative real-time PCR (qRT-PCR) experiment was performed using a SYBR Premix Ex Taq Kit (Takara, Tokyo, Japan) on a Real-Time Fluorescent Quantitative PCR Instrument (Bio-Rad, CA, United States) according to the manufacturer’s instructions. GAPDH was used as internal control. The cycling conditions consisted of reverse transcription at 45°C for 10 min, initial denaturation at 95°C for 15 min, then 45 cycles of denaturation at 95°C for 5 s, and annealing/extension at 60°C for 30 s. The data were analyzed using the relative 2^–ΔΔCT^ method.

### RNA-seq Analysis

The RNA-seq technique was performed to assess the gene expression profiling in SW480 cells with DUSP9 stable knockdown and SW480-shControl cells. The procedure was performed as previously described (20). Trizol Kit (Promega, United States) was used to extract the total RNA of the above samples. The cDNA fragments were purified and enriched by PCR to construct the cDNA library. Finally, the cDNA library was sequenced on the Illumina sequencing platform (IlluminaHiSeq^TM^ 2500). All the clean reads were mapped to reference genome using TopHat. Cufflinks package was used to estimate expression profile and to reconstruct transcript based on genome annotation. Next, the transcripts were merged by cuffmerge. In the last, transcript expression was estimated using cuffquant and cuffnorm. The threshold of the *P*-value in multiple tests was determined by the false discovery rate (FDR). A threshold of the FDR ≤0.05 was used to judge the significance of gene expression differences.

### Bisulfite Sequencing

Bisulfite sequencing (BSE) was performed as previously described (21). Briefly, CRC and normal intestinal mucosa samples were digested with proteinase K to extract sample DNA, which was subject to the bisulfite reaction using an EpiTect Bisulfte kit (Qiagen, Germany) and quantified using a NanoDrop instrument (Thermo Fisher Scientific, Rockford, IL, United States). Modified DNA (40 ng/reaction) was amplified by PCR (using 0.2 μM of each primer, 2 units of HotStart Taq DNA polymerase, and 0.2 mM of each dNTP per reaction). Cycling programs (Applied Biosystems, Life Technologies, United States) were 95°C for 5 min, then 40 cycles of 95°C for 10 s, 60°C for 30 s, and 72°C for 20 s, followed by a 5-min incubation at 72°C. PCR products were examined after gel electrophoresis in 1.5% agarose to confirm that a single band was obtained. Modified DNA was detected by gel electrophoresis. In addition, the BLAST program of the National Center for Biotechnology Information was used to identify the sequence homologies.

### Dual-Luciferase Reporter Assay

The dual-luciferase reporter assay was performed as previously described (22). Briefly, the wild and mutant DUSP9 3′-UTR were inserted into the reporter plasmid. Cells were co-transfected with plasmids and miR-1246 mimics or negative control using Lipofectamine 3000 (Invitrogen). After incubation for 48 h, activity of the Firefly and Renilla luciferase was measured using the Dual-Luciferase Reporter Assay System (Abcam). The luciferase activity was normalized to Renilla fluorescence.

### Statistical Analysis

All statistical analyses were performed using SPSS 17.0 software (SPSS, Chicago, IL). Two-sided Student’s *t*-test was used to examine differences between two groups. Pearson correlation analyses were used to assess the correlations between measured variables. Kaplan–Meier survival curve was plotted for OS and RFS and compared by log-rank test. In this study, we adopted WebGestalt (an online tool) to perform gene set enrichment analysis (GSEA). *P*-values < 0.05 were considered statistically significant.

## Results

### DUSP9 Is Downregulated in CRC Tissues and Associated With Tumor Progression and Poor Prognosis

**FIGURE 1:**
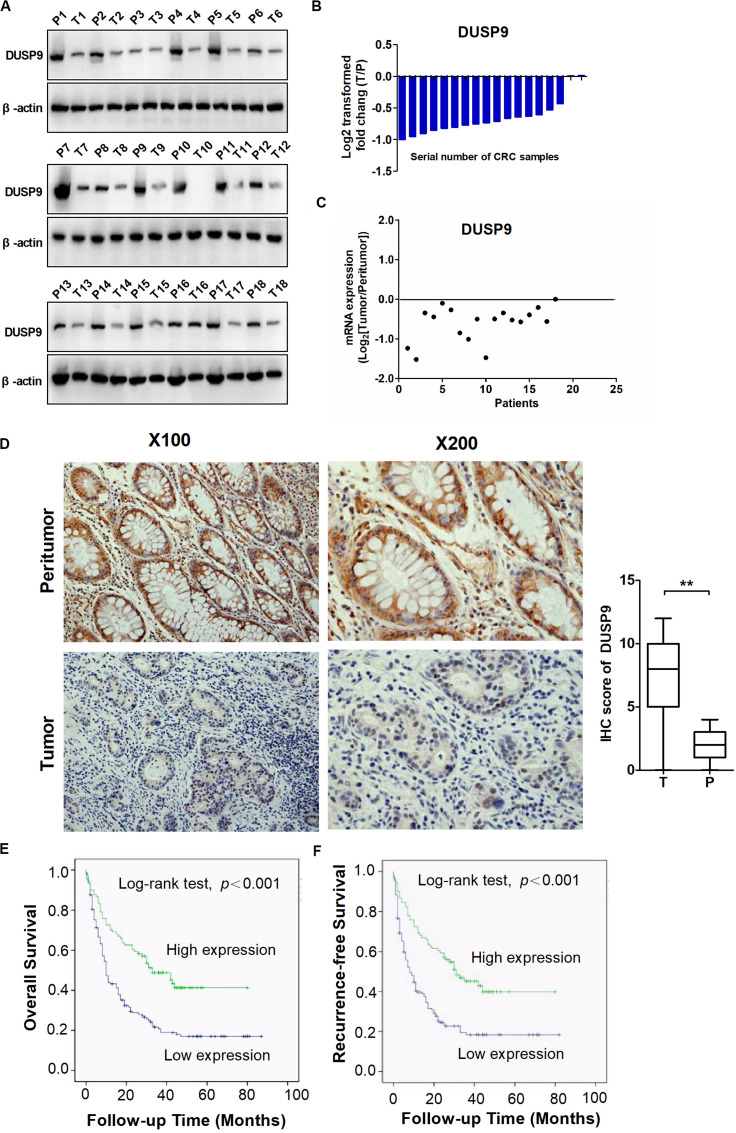
DUSP9 is downregulated in CRC tissues and associated with tumor progression and poor prognosis. **(A)** The protein expression levels of DUSP9 in 18 paired CRC and peritumor tissues were performed by western blot. T, tumor; P, peritumor. **(B)** Protein quantitative results of western blot. **(C)** The mRNA expression levels of DUSP9 in 18 paired CRC and peritumor tissues were performed by qRT-PCR analysis. **(D)** Immunohistochemical (IHC) staining analysis of DUSP9 expression in paired CRC and peritumor tissues (*n* = 245). Scale bars, 50 μm. **P < 0.01. **(E,F)** Kaplan–Meier survival curves for overall survival (OS) and recurrence-free survival (RFS) stratified by DUSP9 expression in 245 tumor issues from CRC patients.

To assess the role of DUSP9 in human CRC, we first evaluated the protein expression levels of DUSP9 in 18 paired CRC tissues from one cohort of 245 patients by western blot. Our results demonstrated that DUSP9 was significantly downregulated in CRC tissues compared with peritumor tissues (Figure 1A). The results of protein quantification are shown in Figure 1B. In addition, quantitative real-time reverse transcription (qRT-PCR) analysis showed that DUSP9 was downregulated in CRC tissues at mRNA level (Figure 1C). As shown in Figure 1D, immunohistochemical (IHC) staining analysis in CRC tissues from one cohort of 245 patients further confirmed the downregulation of DUSP9 at the protein level in CRC tissues compared with paired peritumor tissues. Finally, we explored the clinical relevance of DUSP9 protein expression. Kaplan–Meier survival analysis showed that the overall survival (OS) and recurrence-free survival (RFS) of patients with low protein expression of DUSP9 were significantly shorter than that of patients with high protein expression of DUSP9 (Figures 1E,F). Moreover, low DUSP9 protein expression in CRC was closely associated with tumor size, depth of invasion, and advanced TNM stage, indicating that DUSP9 may be involved in the progression of CRC (Table 1). In addition, DUSP9 protein expression, depth of invasion, and TNM stage were found to be associated with OS and disease-free survival (DFS) of these patients in univariate survival analysis. Multivariate analysis indicated that DUSP9 expression could be a prognostic factor for OS and DFS of patients with CRC after adjusting for gender, age at diagnosis, depth of invasion, and TNM stage, indicating that DUSP9 may be an independent prognostic factor for CRC (Supplementary Tables 1, 2).

### DUSP9 Promoter Hypermethylation Contributes to DUSP9 Silencing in Human CRC

**FIGURE 2:**
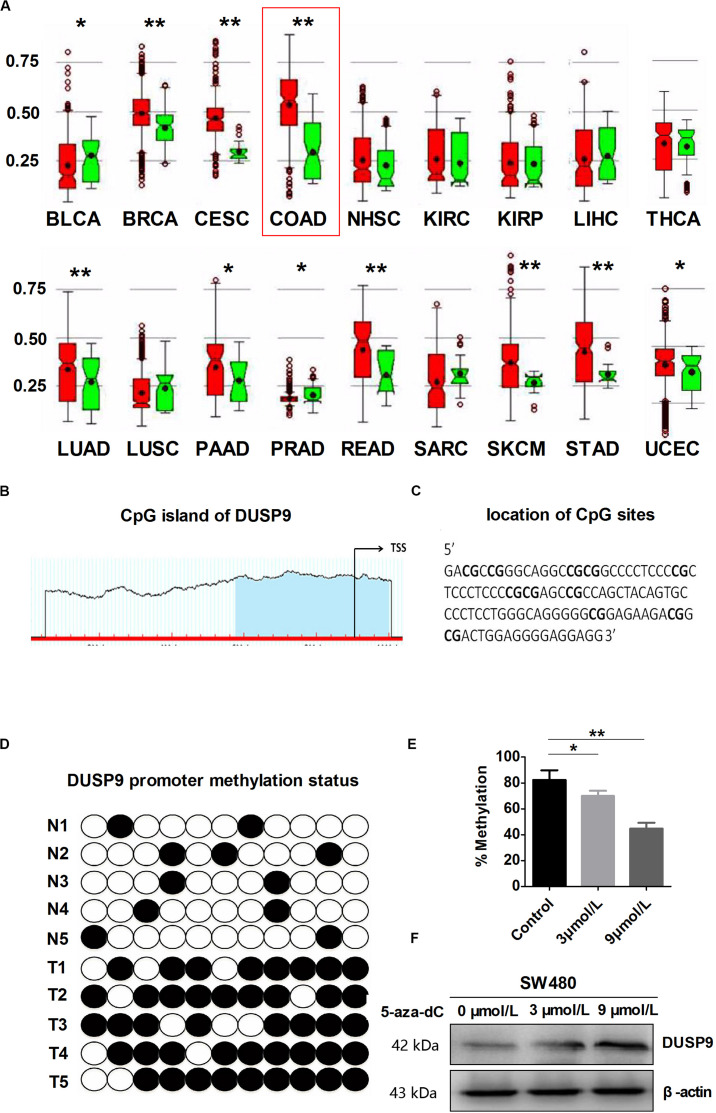
DUSP9 promoter hypermethylation contributes to DUSP9 silencing in human CRC. **(A)** MethHC (the human pan-cancer methylation database) was used to predict the methylation status of DUSP9 gene promoter in a variety of cancers, including bladder urothelial carcinoma (BLCA), breast invasive carcinoma (BRCA), cervical squamous cell carcinoma and endocervical adenocarcinoma (CESC), colon adenocarcinoma (COAD), head and neck squamous cell carcinoma (NHSC), kidney renal clear cell carcinoma (KIRC), kidney renal papillary cell carcinoma (KIRP), liver hepatocellular carcinoma (LIHC), thyroid carcinoma (THCA), lung adenocarcinoma (LUAD), lung squamous cell carcinoma (LUSC), pancreatic adenocarcinoma (PAAD), prostate adenocarcinoma (PRAD), rectum adenocarcinoma (READ), sarcoma (SARC), skin cutaneous melanoma (SKCM), stomach adenocarcinoma (STAD), and uterine corpus endometrial carcinoma (UCEC). *P < 0.05, **P < 0.01. Red represents tumor and green represents normal tissue. **(B)** MethHC was used to predict the CpG island of DUSP9, which extends from –58 to –480 from TSS. Each red tick mark represents one CpG site. The arrows indicate the TSS. CpG island prediction criteria used: island size >100, GC percent >50.0, Obs/Exp >0.6. **(C)** To determine the methylation level, BSP was carried out on 11 CpG sites extending from –230 to –337 from TSS (underlined). **(D)** Bisulfite sequencing evaluation of CpG island methylation of the 11 CpG sites of DUSP9 promoter in CRC (*T* = 12) and normal intestinal mucosa (*N* = 12). White spots, unmethylated CpG; black spots, methylated CpG. **(E)** Bisulfite sequencing was carried out on 11 CpG sites in SW480 cells following treatment with 5-aza-dC for 72 h to determine the methylation level. *P < 0.05, **P < 0.01. **(F)** Treatment with 5-aza-dC can lead to DNA demethylation and restore DUSP9 expression in SW480 cells. TSS, transcription start site.

In order to further explore the reason for the decrease of DUSP9 in CRC, we used MethHC (the human pan-cancer methylation database) to predict the methylation status in DUSP9 gene promoter. The results showed that DUSP9 was hypermethylated in a variety of cancers, such as colon adenocarcinoma (COAD), bladder urothelial carcinoma (BLCA), breast invasive carcinoma (BRCA), cervical squamous cell carcinoma and endocervical adenocarcinoma (CESC), lung adenocarcinoma (LUAD), pancreatic adenocarcinoma, etc. (Figure 2A). Based on the criteria and algorithm described by Li and Dahiya (23), the DUSP9 promoter contains a large CpG island near the transcription start site (Figure 2B). Of note, 11 CpG sites in the DUSP9 promoter were involved in this study for BSE analysis (Figure 2C). The results showed that in normal intestinal mucosa (*n* = 12), the DUSP9 promoter showed hypomethylation status (average methylation level was 15.4%), while in CRC (*n* = 12), the DUSP9 promoter showed hypermethylation status (average methylation level was 87.4%, *P* < 0.01) (Figure 2D). According to previous reports, 5-aza-2′-deoxycytidine (5-aza-dC) is typically used to activate methylated genes by promoter demethylation (24, 25). Thus, we treated SW480 cells with 5-aza-dC and examined DUSP9 promoter methylation and protein expression changes to further determine the relationship between the methylation level and expression level of DUSP9 in CRC. The results showed that with the increase of 5-aza-dC concentration, the methylation level of DUSP9 decreased (Figure 2E), while the protein expression level increased gradually (Figure 2F). This suggests that promoter hypermethylation is one of the reasons for low expression of DUSP9 in CRC.

### DUSP9 Silencing Is Also Mediated by the Upregulation of miR-1246

**FIGURE 3:**
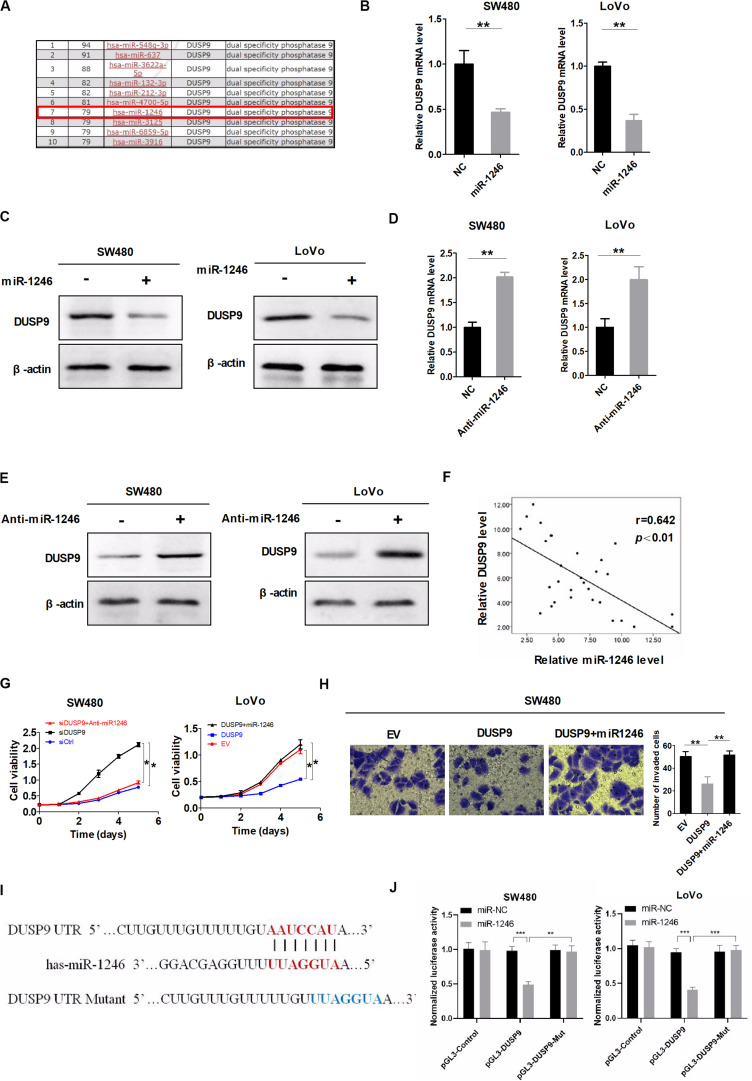
DUSP9 silencing is also mediated by the upregulation of miR-1246. **(A)** The top 10 predicted miRNAs targeting DUSP9 were identified using microRNA Data Integration Portal (mirDIP)-based target prediction programs. **(B)** qRT-PCR and **(C)** western blot analysis for DUSP9 expression in SW480 and LoVo cells transfected with the miR-1246 mimics. **(D)** qRT-PCR and **(E)** western blot analysis for DUSP9 expression in SW480 and LoVo cells transfected with the miR-1246 inhibitor (anti-miR1246). **(F)** The correlation between the mRNA levels of DUSP9 and miR-1246 was determined. **(G)** MTS cell viability assays in SW480 and LoVo cells with treatment as indicated. **(H)** Transwell matrigel invasion assays in SW480 cells with treatment as indicated. The number of invaded cells was per microscopic field. Scale bars, 50 μm. *P < 0.05, **P < 0.01, and ***P < 0.005.

Accumulating evidence has suggested that microRNA (miRNA) is critical to the regulation of gene-expression network and is frequently dysregulated in many types of cancers. In this study, microRNA Data Integration Portal (mirDIP)-based target prediction programs were used to identify the potential miRNAs involved in the downregulation of DUSP9 in CRC. The top 10 predicted miRNAs targeting DUSP9 were listed in Figure 3A. Real-time PCR and western blot showed that miR-1246 remarkably reduced DUSP9 expression in SW480 and LoVo cells (Figures 3B,C). On the contrary, micro-1246 inhibition can elevate the expression of DUSP9 in both mRNA and protein levels (Figures 3D,E). Moreover, a significant negative correlation (*r* = −0.642, *P* < 0.01) between the mRNA expression of miR-1246 and DUSP9 was found in tumor tissues from 30 CRC patients (Figure 3F). In addition, miR-1246 mimics attenuated the ability of DUSP9 to inhibit the proliferation of CRC cells, whereas miR-1246 inhibition decreased the proliferation by DUSP9 knockdown in CRC cells (Figure 3G). Moreover, transwell invasion experiment revealed that miR-1246 mimics attenuated the ability of DUSP9 to inhibit the invasion of CRC cells (Figure 3H). Altogether, these results suggest that miR-1246 leads to the downregulation of DUSP9 and promotes the proliferation and invasion of CRC cells by inhibiting DUSP9. To further investigate the direct relationship between miR-1246 and DUSP9, we conducted dual luciferase reporters containing the 3′UTR of DUSP9 and the mutant type 3′UTR of DUSP9 (Figure 3I). The results showed that miR-1246 significantly reduced the luciferase activity of DUSP9 wild-type reporters compared with mutant-type reporters (Figure 3J). Altogether, these results suggest that miR-1246 leads to the downregulation of DUSP9 and promotes the proliferation and invasion of CRC cells by inhibiting DUSP9.

### DUSP9 Inhibits Proliferation, Migration, Invasion, and Epithelial–Mesenchymal Transition of CRC Cells *in vitro*

**FIGURE 4:**
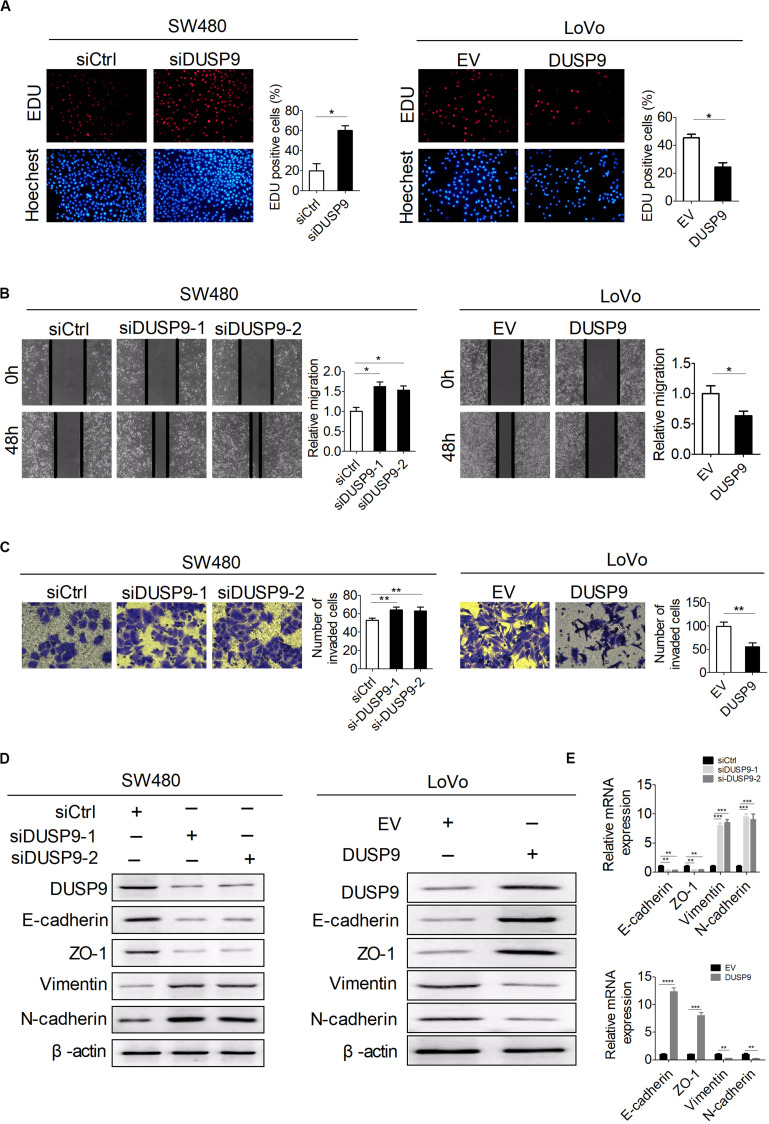
DUSP9 inhibits proliferation, migration, invasion, and epithelial–mesenchymal transition (EMT) of CRC cells *in vitro*. **(A)** The EdU incorporation assay was performed in SW480 and LoVo cells with the indicated treatments (siDUSP9, siRNAs against DUSP9; siCtrl, control siRNA; DUSP9, expression vector encoding DUSP9; EV, empty vector). Scale bars, 50 μm. **(B)** Scratch wound healing assays for cell migration abilities in SW480 and LoVo cells with the indicated treatments. Scale bars, 50 μm. **(C)** Transwell invasion assays for cell invasion abilities in SW480 and LoVo cells with the indicated treatments. Scale bars, 50 μm. **(D,E)** Western blot and RT-PCR analyses for expressions of epithelial–mesenchymal transition (EMT)-related markers in both SW480 and LoVo cells with treatment as indicated. *P < 0.05, **P < 0.01, ***P < 0.001, ****P < 0.0001.

Next, a variety of *in vitro* assays were carried out with loss of function or gain of function of DUSP9 to evaluate the potential role of DUSP9 on CRC cell functions. The number of 5-ethynyl-2′-deoxyuridine (EdU) incorporation was significantly increased in SW480 cells with DUSP9 knockdown but was markedly decreased in LoVo cells with DUSP9 overexpression compared with controls (Figure 4A). The scratch wound healing assays showed that knockdown of DUSP9 significantly promoted the migratory ability of SW480 cells. In contrast, DUSP9 overexpression remarkably inhibited the migratory ability of LoVo cells (Figure 4B). Accordingly, the transwell invasion assay showed that DUSP9 overexpression significantly impaired the invasion of LoVo cells. However, knockdown of DUSP9 in SW480 cells acted the opposite way (Figure 4C). It is well established that the epithelial–mesenchymal transition (EMT) plays a key role in tumor metastasis by increasing cell mobility and reducing cell–cell contact. However, the relationship between DUSP9 and EMT has not been reported yet. Thus, we further investigated whether EMT is involved in DUSP9-mediated invasion and metastasis of CRC cells. According to previous studies, N-cadherin and vimentin were commonly used mesenchymal markers and E-cadherin and zonula occludens-1 were commonly used epithelial markers (26, 27). Thus, we detected the expression level of these four molecules by western blot analysis and qRT-PCR. The results showed that mesenchymal markers (N-cadherin and vimentin) were significantly increased and epithelial markers (E-cadherin and zonula occludens-1) were remarkably decreased when DUSP9 was knocked down in SW480 cells. However, the opposite effect was observed following DUSP9 overexpression in LoVo cells (Figures 4D,E). These findings indicated that it inhibits proliferation, migration, invasion, and EMT of CRC cells *in vitro*.

### DUSP9 Suppresses Tumor Growth *in vivo*

**FIGURE 5:**
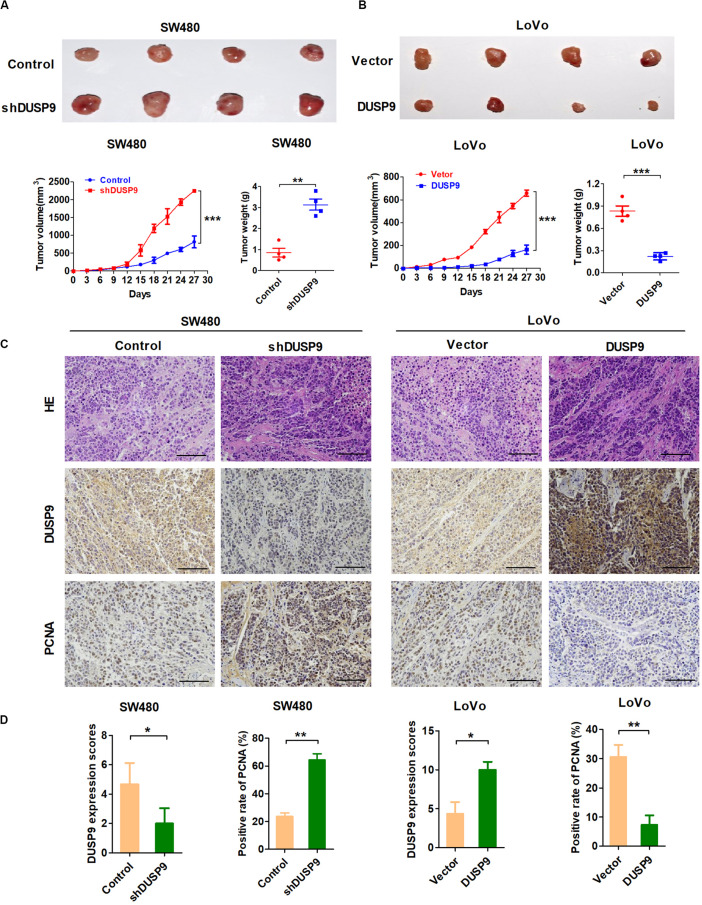
DUSP9 suppresses tumor growth *in vivo.* **(A)** DUSP9 knockdown promoted growth of SW480 xenograft tumors, which were generated by injecting SW480-shDUSP9 cells or SW480-Control cells. The growth of xenograft tumors was measured by volume, and the tumor weight was measured. **(B)** DUSP9 overexpression inhibited growth of LoVo xenograft tumors, which were generated by injecting LoVo cells overexpressing DUSP9 or carrying a control vector. The growth of xenograft tumors was measured by volume, and the tumor weight was measured. **(C,D)** HE, IHC, and PCNA staining of the xenograft tumors are shown. Scale bars, 50 μm. *P < 0.05, **P < 0.01, and ***P < 0.005.

In order to further verify the tumorigenicity of DUSP9 *in vivo*, we constructed a tumor model of nude mice using SW480 and LoVo CRC cell lines with stable overexpression or knockdown of DUSP9. These cells were then transplanted into nude mice to test their tumorigenicity *in vivo*. Subcutaneous tumor growth was monitored every 3 days, and these mice were euthanized after 30 days. Tumors derived from SW480 cells with stable DUSP9 knockdown showed an increased growth rate and less net weight at the fourth week when compared with control mice (Figure 5A). In contrast, the tumor growth rate was slower, and the average tumor weight was significantly reduced in mice inoculated with LoVo cells with stable DUSP9 overexpression at the fourth week when compared with control mice (Figure 5B).

Moreover, when compared with controls, the xenografts developed from SW480 cells with DUSP9 stable knockdown exhibited a significant increase of positive PCNA staining. However, overexpression of DUSP9 exhibited a considerable decrease of positive PCNA staining in xenografts developed from LoVo cells (Figures 5C,D). In conclusion, the above results further confirmed the antitumor effect of DUSP9 on the progression of CRC.

### Transcriptome Profiling Studies Revealed That Erk Signaling Was Involved in the Tumor Progression Mediated by DUSP9 Silencing

**FIGURE 6:**
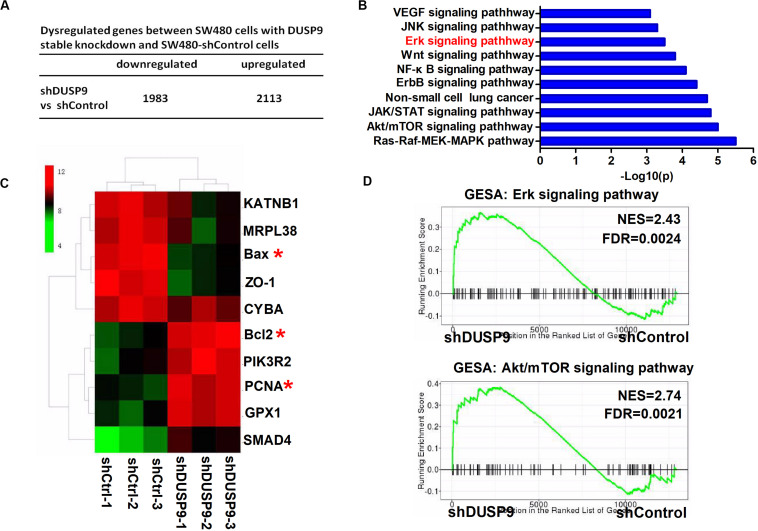
Transcriptome profiling studies revealed that Erk signaling was involved in the tumor progression mediated by DUSP9 silencing. **(A)** Dysregulated genes between SW480 cells with DUSP9 stable knockdown and SW480-shControl cells. Of the 4096 dysregulated genes between two groups, 2113 genes were upregulated (fold change ≥2) and 1983 genes were downregulated (fold change ≥2). **(B)** Top 10 KEGG pathway enriched by the differentially expressed genes. **(C)** Cluster analysis of DEGs annotated in pathways associated with cell proliferation-related and cell apoptosis-related markers. **(D)** GSEA (gene set enrichment analysis) shows that DUSP9 knockdown will lead to the activation of Erk and Akt/mTOR signaling pathway. *P < 0.05.

To determine the biological function of DUSP9 in CRC, we performed RNA-seq in SW480 cells upon DUSP9 knockdown followed by a differential expression analysis to determine which genes are significantly deregulated. Of the 4096 dysregulated genes between two groups, 2113 genes were upregulated (fold change ≥2) and 1983 genes were downregulated (fold change ≥2) (Figure 6A). Part of representative dysregulated genes between SW480 cells with DUSP9 stable knockdown and SW480-shControl cells are listed in Supplementary Table 3. In order to explore the role of DURP9 in tumor progression, we performed KEGG pathway analysis of these DEGs between two groups, and the results showed that many tumor growth and metastasis-related pathways, such as Erk, JNK, Wnt, Akt/mTOR, and ErbB signaling pathways, were significantly enriched in the DUSP9 knockdown group. This suggests that DUSP9 knockdown can activate tumor growth and metastasis-related pathways (Figure 6B). A hierarchical cluster of DEGs is partially shown in Figure 6C. As shown in the heatmap, cell proliferation-related markers (bcl2 and PCNA) were significantly increased and cell apoptosis-related markers (such as Bax) were remarkably decreased in shDUSP9 SW480 cells than in control cells, indicating that DUSP9 knockdown significantly promotes the progression of CRC. Moreover, GSEA also showed that downregulation of DUSP9 led to the activation of Erk and Akt/mTOR signaling pathway (Figure 6D).

### Activation of the Erk Pathway Is Involved in the DUSP9 Silencing-Mediated Tumor Growth of CRC

Because Erk pathway activation plays a key role in tumor growth and metastasis, we hypothesized that the oncogenic phenotype induced by DUSP9 silencing may be associated with activation of the Erk pathway. To test this possibility, we treated SW480 cells or LoVo cells with the specific Erk signaling inhibitor PD98059 or Erk signaling activator Curcumin.

**FIGURE 7:**
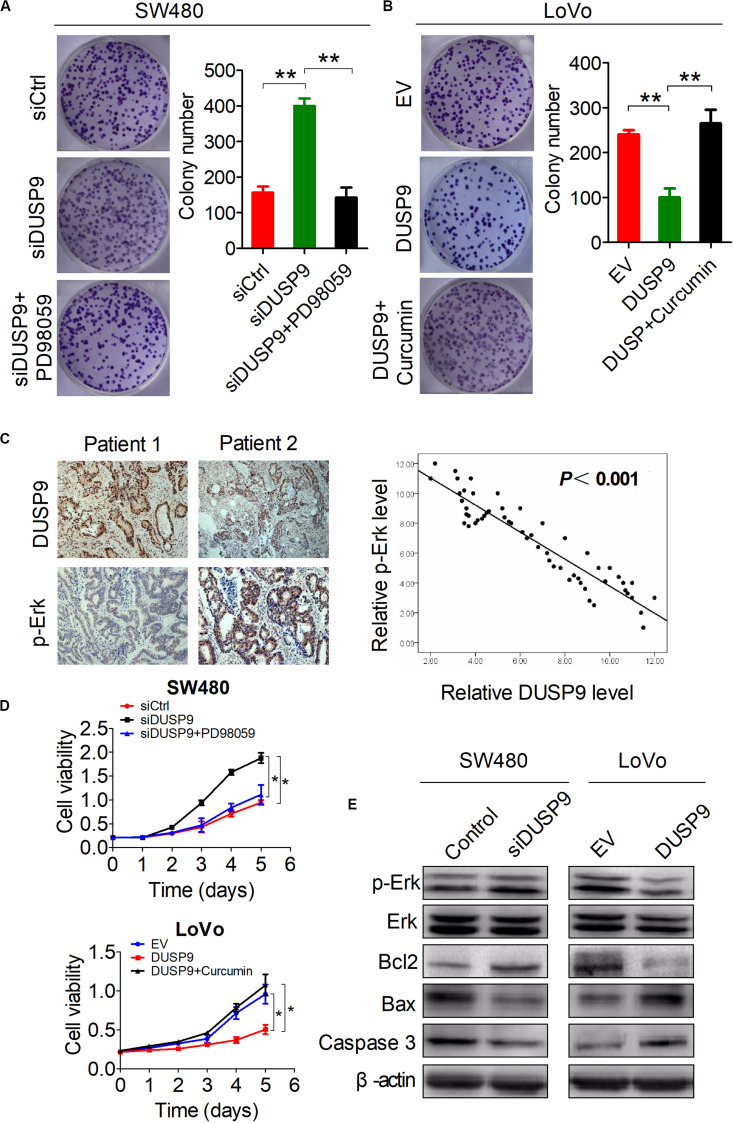
Activation of the Erk pathway is involved in the DUSP9 silencing-mediated tumor growth of CRC. **(A,B)** Colony formation assays for cell growth ability of HCC cells upon treatment with the specific Erk signaling inhibitor PD98059 or activator Curcumin. **(C)** Left panel: representative IHC images of DUSP9 and p-Erk in tumor tissues of CRC. Right panel: relationship between the expression of DUSP9 and p-Erk was analyzed based on IHC staining. Scale bars, 50 μm. **(D)** MTS assays for cell growth ability of HCC cells upon treatment with the specific Erk signaling inhibitor PD98059 or activator Curcumin. **(E)** Western blot analyses for expressions of cell apoptosis-related markers in both SW480 and LoVo cells with treatment as indicated. *P < 0.05, **P < 0.01.

As shown in Figures 7A,B, PD98059 treatment significantly decreased the growth of SW480 cells induced by DUSP9 knockdown, whereas Curcumin treatment significantly increased the growth of LoVo cells suppressed by DUSP9 overexpression. IHC studies showed a significant negative correlation between the IHC scores of DUSP9 and p-Erk (*P* < 0.001) (Figure 7C). The results of MTS cell viability assay also showed that PD98059 treatment significantly decreased the growth of SW480 cells induced by DUSP9 knockdown, whereas Curcumin treatment significantly increased the growth of LoVo cells suppressed by DUSP9 overexpression, indicating that Erk activation is involved in the DUSP9 silencing-mediated tumor growth of CRC (Figure 7D). We also examined the effect of DUSP9 on cell apoptosis-related molecules. As expected, western blot analysis showed that the protein expression of p-Erk and two major cell apoptosis-related molecules Bax and caspase3 was decreased in SW480 cells with DUSP9 knockdown, while overexpression of DUSP9 in LoVo cells had the opposite effect (Figure 7E), confirming the role of DUSP9 in promoting cell apoptosis progression in CRC cells by inhibiting the Erk pathway.

## Discussion

**FIGURE 8:**
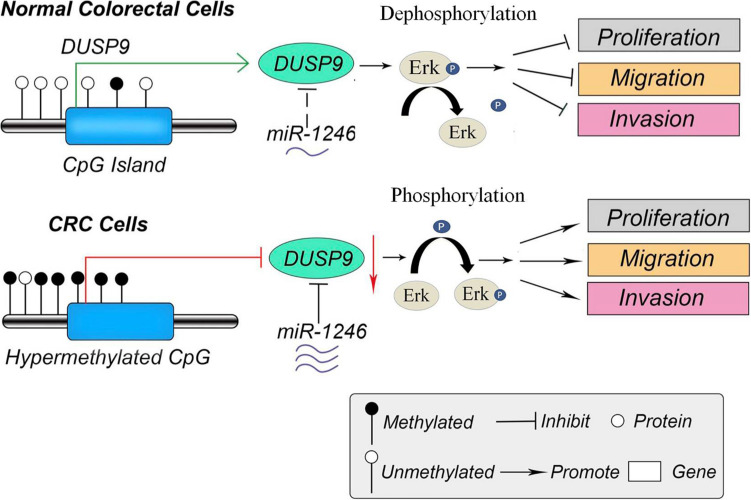
The schematic diagram of DUSP9 in CRC. In normal colorectal cells, the promoter of DUSP9 is hypomethylated, and the expression level of DUSP9 is relatively high, which inhibits cell proliferation, migration, and invasion by dephosphorylating ERK. However, the hypermethylation status of CpG island in promoter of DUSP9 could lead to a significant decrease of DUSP9 expression levels in human CRC. Moreover, the increased miR-1246 in CRC further reduced the expression level of DUSP9, which promoted tumor cell proliferation, migration, and invasion by phosphorylating ERK. CRC, colorectal cancer.

Recently, low expression levels of DUSP9 were reported in a variety of cancers, such as gastric cancer, liver cancer, and renal cancer. In the present study, we systematically investigated the functional role of DUSP9 in CRC and found that DUSP9 was significantly downregulated in tumor tissues compared with peritumor tissues. Moreover, low expression of DUSP9 was correlated with survival time and poor prognosis of patients with CRC. Further statistical analysis revealed that low DUSP9 expression level in CRC was closely associated with tumor size, depth of invasion, and advanced TNM stage, indicating that DUSP9 may be involved in the progression of CRC. Mechanistically, low expression of DUSP9 in CRC was caused by the upregulation of miR-1246. In addition, promoter hypermethylation is another reason for the downregulation of DUSP9 in CRC. Functional study revealed that DUSP9 inhibited tumor migration, invasion, and metastasis both *in vitro* and *in vivo*. Moreover, activation of the Erk signaling is involved in the DUSP9 silencing-mediated tumor growth of CRC (Figure 8).

It has been reported that DUSP9 could affect cell proliferation and participated in the regulation of tumor progression. To our knowledge, this is the first study to report the clinical significance of DUSP9 in CRC. To determine the biological function of DUSP9 in CRC, we performed RNA-seq analysis of gene profiling between CRC cells with DUSP9 knockdown and control cells, and the results revealed that DUSP9 inhibited proliferation and metastasis-related pathways. Next, a series of *in vitro* and *in vivo* experiments confirmed that DUSP9 significantly inhibited the proliferation, invasion, and metastasis of CRC cells. However, the molecular mechanism of DUSP9 inhibiting CRC progression has not been fully elucidated. In gastric cancer, low expression level of DUSP9 has been found to be linked with the increased JNK activity and decreased apoptosis, suggesting that DUSP9 may inhibit the proliferation of gastric cancer cells through JNK signaling pathway (21). In CRC, DUSP9 is known to inactivate many members of the mitogen-activated protein (MAP) kinase superfamily (such as SAPK, MAPK, and p38) by dephosphorylating both the phosphotyrosine and phosphoserine/threonine residues (28–30). The growth speed of the CRC cells with DUSP9 overexpression was slow and may even be likened to a growth arrest (10, 31). In addition, Wu et al. (10) demonstrated that low expression of the DUSP9 was an independent indicator for poor prognosis of patients with clear cell renal cell carcinomas (ccRCCs). Moreover, enhanced expression of DUSP9 in malignant tumor cells led to microtubule disruption, cell death, and tumor inhibition (9, 32, 33). In this study, we found that Erk signaling activation was involved in the tumor progression mediated by DUSP9 silencing. Therefore, DUSP9 may synergistically inhibit the progression of CRC through a variety of mechanisms, which needs to be further verified in future research.

MicroRNAs are a class of important small molecule non-coding RNA, which regulates target mRNAs at the post-transcriptional level by binding to their 3′-untranslated regions (3-UTRs), resulting in translation inhibition or degradation of mRNAs (34). MiR-1246 is a frequently upregulated carcinogenic factor and is involved in the invasion, metastasis, and chemoresistance in a variety of cancers, including CRC. For instance, Yang et al. (35) demonstrated that miR-1246 promotes metastasis and invasion of lung cancer cells by regulating Wnt/β-catenin pathway. Lin et al. (36) reported that miR-1246 was significantly upregulated in oral squamous cell carcinoma (OSCC) tissues and enhanced the stemness hallmarks, which closely associated with cancer relapse and metastasis. In CRC, miR-1246 was also found to be significantly increased in CRC tissues and functioned as a tumor-promoting factor through inducing cell proliferation, migration, invasion, and chemoresistance (37, 38). Moreover, some specific exosomal miRNAs, including miR-1246, were identified as a biomarker for metastatic CRC (39–41). In addition, Peng et al. (42) demonstrated that the miR-1246/SPRED2/MAPK axis played an important role in the progression of CRC. Consistently, our present study showed that miR-1246 was involved in the downregulation of DUSP9 in CRC. Furthermore, we found that miR-1246 promoted the growth and invasion of CRC cells by inhibiting DUSP9. Therefore, miR-1246/DUSP9 axis might be a promising strategy for CRC treatment.

Methylation of CpG island in the promoter region of tumor suppressor genes can promote the initiation of many cancers, including CRC. Moreover, the *de novo* methylation of genes seems to be a common event in most malignancies (43–46). In many types of cancer, hypermethylation of CpG island in the promoter of tumor suppressor genes is an important event. It can affect genes related to cell cycle, apoptosis, DNA repair, angiogenesis, etc. (21, 47, 48). In the present study, we used MethPrimer software to predict the methylation status in the DUSP9 gene promoter and found that DUSP9 was hypermethylated in a variety of cancers, such as COAD, BLCA, BRCA, LUAD, pancreatic adenocarcinoma, et al. Furthermore, BSE analysis revealed the hypermethylation status of CpG island in the promoter of DUSP9, which further led to a significant decrease of DUSP9 expression levels in human CRC. As far as we know, this is the first study using BSE analysis for methylation status analysis of DUSP9 in clinical human CRC samples.

## Conclusion

In conclusion, this work contributes to progress the knowledge in the field of CRC biomarkers. This finding suggests that DUSP9 may serve as a potential CRC biomarkers. In addition, therapeutic intervention to increase the expression or activity of DUSP9 may be a potential therapeutic target for CRC treatment in the future (Figure 8).

## Data Availability Statement

The datasets presented in this study can be found in online repositories. The names of the repository/repositories and accession number(s) can be found below: https://www.ncbi.nlm.nih.gov/bioproject/, accession: PRJNA659790.

## Ethics Statement

The studies involving human participants were reviewed and approved by the Medical Ethics Committee of PLA General Hospital. The patients/participants provided their written informed consent to participate in this study. The animal study was reviewed and approved by the Medical Ethics Committee of PLA General Hospital.

## Author Contributions

ZQ and QW designed the study. ZQ and NL wrote the manuscript. QH polished the article. TS and HX performed the experiment. ZQ, QW, and TX analyzed the data. XW helped revised the manuscript. All authors read and approved the final manuscript.

## Conflict of Interest

The authors declare that the research was conducted in the absence of any commercial or financial relationships that could be construed as a potential conflict of interest.

## Abbreviations

DUSP9: dual-specificity phosphatase 9
CRC: colorectal cancer
BSE: bisulfite sequencing
MKP-4: mitogen-activated protein kinase phosphatase 4
NAFLD: non-alcoholic fatty liver disease
NSCLC: non-small cell lung cancer
qRT-PCR: quantitative real-time reverse transcription PCR
OS: overall survival
DFS: disease-free survival
DEGs: differently expressed genes
EMT: epithelial–mesenchymal transition
COAD: colon adenocarcinoma
BLCA: bladder urothelial carcinoma
BRCA: breast invasive carcinoma
CESC: cervical squamous cells carcinoma
LUAD: lung adenocarcinoma
SCC: squamous cell carcinoma
ccRCCs: clear cell renal cell carcinomas
3 ′ -UTR: 3 ′ -untranslated regions
miRNAs: microRNAs
OSCC: oral squamous carcinoma.
