# Supplementary material for: Downregulation of DUSP9 Promotes Tumor Progression and Contributes to Poor Prognosis in Human Colorectal Cancer
Source: Front Oncol. 2020 Sep 23;10:547011. doi: 10.3389/fonc.2020.547011 (PMC7538709; doi:10.3389/fonc.2020.547011)
Supplement: Supplementary file 1 [file Table_1.DOCX]

**Supplementary Table 1. Association of DUSP9 and clinical factors with overall survival**

|  | |  | Unadjusted HR ^*^ (95% CI) | *P* | Adjusted HR^†^ (95% CI) | *P* |
| --- | --- | --- | --- | --- | --- | --- |
| DUSP9 expression | |  | 0.002(0.000-0.01) | **0.000** | 0.004(0.001-0.014) | 0.000 |
| Gender | |  | 1.506(0.171-13.24) | 0.712 | - | - |
| Age at diagnosis | |  | 1.030(0.093-11.45) | 0.981 | - | - |
| Tumor site | |  | 2.370(0.272-20.582) | 0.436 | - | - |
| Tumor size | |  | 2.690(0.33-22.21) | 0.357 | - | - |
| Depth of invasion | |  | 6.890(2.27-20.94) | **0.001** | 15.573(5.882-41.232) | 0.000 |
| TNM stage |  | | 60.330(5.31-686.04) | **0.001** | 129.013(16.326-1019.48) | 0.000 |

^*^Hazard ratios in univariate models

^†^Hazard ratios in multivariable models

Abbreviations: HR, hazard ratio; 95% CI, 95% confidence interval.
