# Supplementary material for: Downregulation of DUSP9 Promotes Tumor Progression and Contributes to Poor Prognosis in Human Colorectal Cancer
Source: Front Oncol. 2020 Sep 23;10:547011. doi: 10.3389/fonc.2020.547011 (PMC7538709; doi:10.3389/fonc.2020.547011)
Supplement: Supplementary file 2 [file Table_2.DOCX]

**Supplementary Table 2. Association of DUSP9 and clinical factors with disease-free survival.**

|  | |  | Unadjusted HR ^*^ (95% CI) | *P* | Adjusted HR^†^ (95% CI) | *P* |
| --- | --- | --- | --- | --- | --- | --- |
| DUSP9 expression | |  | 0.006(0.002-0.020) | **0.000** | 0.006(0.002-0.022) | 0.000 |
| Gender | |  | 1.382(0.159-11.99) | 0.769 | - | - |
| Age at diagnosis | |  | 0.638(0.057-7.107) | 0.715 | - | - |
| Tumor site | |  | 1.310(0.162-10.604) | 0.800 | - | - |
| Tumor size | |  | 2.380(0.306-18.501) | 0.407 | - | - |
| Depth of invasion | |  | 7.136(2.455-20.741) | **0.000** | 11.269(4.865-26.102) | 0.000 |
| TNM stage |  | | 71.186(6.343-798.915) | **0.001** | 102.769(13.304-793.859) | 0.000 |

^*^Hazard ratios in univariate models

^†^Hazard ratios in multivariable models

Abbreviations: HR, hazard ratio; 95% CI, 95% confidence interval.
