# Supplementary material for: Downregulation of DUSP9 Promotes Tumor Progression and Contributes to Poor Prognosis in Human Colorectal Cancer
Source: Front Oncol. 2020 Sep 23;10:547011. doi: 10.3389/fonc.2020.547011 (PMC7538709; doi:10.3389/fonc.2020.547011)
Supplement: Supplementary file 3 [file Table_3.DOCX]

**Supplementary table 3. List of representative dysregulated genes between SW480 cells with DUSP9 stable knockdown and SW480-shControl cells.**

| Gene name | Description | Functions | Significance (Pvalue) |
| --- | --- | --- | --- |
| KRT7 | Keratin 7 | The protein encoded by this gene is a member of the keratin gene family. | Yes (5.15E-80) |
| CD164 | CD164 molecule | Regulates the proliferation, adhesion and migration of hematopoietic progenitor cells | Yes (10.03E-42) |
| ITGA5 | Integrin subunit alpha 5 | Cell surface adhesion and signaling | Yes (1.46E-51) |
| GOLM1 | Golgi membrane protein 1 | Sorting and modification of proteins exported from the endoplasmic reticulum | Yes (4.50E-26) |
| NBL1 | NBL1, DAN family BMP antagonist | Play an important role during growth and development | Yes (1.32E-84) |
| ANXA6 | Annexin A6 | Implicated in membrane-related events along exocytotic and endocytotic pathways. | Yes (1.21E-83) |
| CCND1 | Cyclin D1 | Cyclins function as regulators of CDK kinases | Yes (2.67E-31) |
| LMNB2 | Lamin B2 | Regulates nuclear stability, chromatin structure and gene expression. | Yes (4.41E-37) |
| VAMP3 | Vesicle associated membrane protein 3 | This gene is a member of the vesicle-associated membrane protein (VAMP)/synaptobrevin family. | Yes (7.04E-96) |
| DNAJB6 | DnaJ heat shock protein family (Hsp40) member B6 | Protein folding and oligomeric protein complex assembly | Yes (1.85E-41) |
| PKDCC | Protein kinase domain containing, cytoplasmic | Function unknown | Yes (3.00E-83) |
| G3BP2 | G3BP stress granule assembly factor 2 | Ubiquitous expression in brain,thyroid. | Yes (4.44E-48) |
| PSMB10 | Proteasome 20S subunit beta 10 | Broad expression in duodenum adult, large intestine adult | Yes (3.06E-58) |
| MSRB1 | Methionine sulfoxide reductase B1 | The protein encoded by this gene function as repair enzymes that protect proteins from oxidative stress by catalyzing the reduction of methionine-R-sulfoxides to methionines | Yes (2.60E-68) |
| NR2F6 | Nuclear receptor subfamily 2 group F member 6 | Function unknown | Yes (7.35E-54) |
| PDK4 | Pyruvate dehydrogenase kinase 4 | Regulation of glucose metabolism | Yes (8.25E-38) |
| FUOM | fucose mutarotase | Ubiquitous expression in duodenum adult, kidney adultand 27 other tissues | Yes (1.28E-27) |
| OPA1 | OPA1 mitochondrial dynamin like GTPase | The encoded protein localizes to the inner mitochondrial membrane and helps regulate mitochondrial stability and energy output. | Yes (4.35E-31) |
| GCC2 | GRIP and coiled-coil domain containing 2 | The protein encoded by this gene is a peripheral membrane protein localized to the trans-Golgi network. | Yes (4.28E-34) |
| RGPD6 | RANBP2 like and GRIP domain containing 6 | Function unknown | Yes (2.18E-23) |
| SLC2A6 | Solute carrier family 2 member 6 | Hexose transport into mammalian cells is catalyzed by a family of membrane proteins, including SLC2A6, that contain 12 transmembrane domains and a number of critical conserved residues | Yes (8.74E-37) |
| DNM2 | Dynamin 2 | Endocytosis and cell motility | Yes (26.4E-21) |
| ZNF207 | Zinc finger protein 207 | Function unknown | Yes (1.22E-31) |
| RBKS | Ribokinase | Ribose metabolism | Yes (1.45E-42) |
| GAGE1 | G antigen 1 | Function unknown | Yes (4.15E-12) |
| SNAPC3 | Small nuclear RNA activating complex polypeptide 3 | Broad expression in testis, brain and 24 other tissues | Yes (1.80E-43) |
| YIPF6 | Yip1 domain family member 6 | Function unknown | Yes (1.51E-38) |
| ELOVL7 | ELOVL fatty acid elongase 7 | Catalyzes lipidome remodeling essential for human cytomegalovirus replication | Yes (7.01E-11) |
| ABTB1 | Ankyrin repeat and BTB domain containing 1 | Involved in protein-protein interactions. Expression of this gene is activated by the phosphatase and tensin homolog, a tumor suppressor. Alternate splicing results in three transcript variants. | Yes (2.20E-19) |
