# Supplementary material for: Downregulation of DUSP9 Promotes Tumor Progression and Contributes to Poor Prognosis in Human Colorectal Cancer
Source: Front Oncol. 2020 Sep 23;10:547011. doi: 10.3389/fonc.2020.547011 (PMC7538709; doi:10.3389/fonc.2020.547011)
Supplement: Supplementary file 4 [file Table_4.DOCX]

**Supplementary Table 4. Sequences of the Primers**

| Gene | Forward Primer | Reverse Primer |
| --- | --- | --- |
| DUSP9 | 5’-CAGCCGTTCTGTCACCGTC-3’ | 5’-CAAGCTGCGCTCAAAGTCC-3’ |
| E-cadherin | 5’-GCCCCATCAGGCCTCCGTTT-3’ | 5’-ACCTTGCCTTCTTTGTCTTTGTTGGA-3’ |
| N-cadherin | 5’-TGGACCATCACTCGGCTTA-3’ | 5’-ACACTGGCAAACCTTCACG-3’ |
| Vimentin | 5’-CCTGAACCTGAGGGAAACTAA-3’ | 5’-GCAGAAAGGCACTTGAAAGC-3’ |
| ZO-1 | 5’-CACGCAGTTACGAGCAAG-3’ | 5’-TGAAGGTATCAGCGGAGG-3’ |
| GAPDH | 5’-GGAGCGAGATCCCTCCAAAAT-3’ | 5’-GGCTGTTGTCATACTTCTCATGG-3’ |
